# SPIRAL MRI for in vivo lithium-7 imaging: a feasibility study in mice after oral lithium treatment

**DOI:** 10.1038/s41598-023-50841-7

**Published:** 2024-01-05

**Authors:** Tor Rasmus Memhave, Amir Moussavi, Susann Boretius

**Affiliations:** 1 Functional Imaging Laboratory, German Primate Center, Leibniz Institute for Primate Research, Göttingen, Germany; 2 Georg-August Universität Göttingen, Göttingen, Germany; 3 International Max Planck Research School for Neurosciences, Göttingen, Germany

**Keywords:** Preclinical research, Neuroscience

## Abstract

Lithium has been the frontline treatment for bipolar disorder for over 60 years. However, its mode of action and distribution in the brain is still incompletely understood. The primary isotope of lithium, lithium-7 (^7^Li), is a magnetic resonance (MR) active, spin-3/2 nucleus. However, its low MR sensitivity and the small brain size of mice make ^7^Li MR imaging (MRI) difficult in preclinical research. We tested four MRI sequences (FLASH, RARE, bSSFP, and SPIRAL) on lithium-containing phantoms, and bSSFP and SPIRAL on orally lithium-treated adult C57BL/6 mice. ^7^Li MR spectroscopy was acquired weekly at 9.4T to monitor the lithium uptake. The in vivo T1 relaxation time of ^7^Li was estimated in four mice. 4-h SPIRAL ^7^Li MRI was acquired in ten mice at a resolution of 2 × 2 × 3 mm^3^. SPIRAL MRI provided the highest signal-to-noise ratio (SNR) per unit acquisition time and the best image quality. We observed a non-homogeneous distribution of lithium in the mouse brain, with the highest concentrations in the cortex, ventricles, and basal brain regions. Almost no lithium signal was detected in the olfactory bulb and the cerebellum. We showed that in vivo ^7^Li MRI in mice is feasible, although with limited spatial resolution and SNR.

## Introduction

Lithium is a frontline treatment for bipolar disorder that has been used clinically for over 60 years^1^. It has been shown that lithium decreases excitatory neurotransmission and inhibits NMDA receptors^2^. It alters secondary messenger signalling^3–5^ and increases neuroprotection via increased activity of the transcription factor CREB^6,7^. Despite its therapeutic successes on mood disorders, lithium’s potentially complex mode of action in the brain is still not well understood. Of particular interest in this context is that lithium appears to affect individual brain regions differently^8^. This local selectivity may be related to a heterogeneous lithium distribution across the brain, varying enormously between individuals^9,10^. For example, a recent high-field MRI study reported significant clustering of the lithium signal in the left hippocampus in patients with bipolar disorder^10^. In addition, human studies have found increased lithium in the brainstem, white matter, and the limbic system^9–11^. Moreover, *post-mortem* studies on three human brains reported a higher lithium concentration in white matter than in grey matter in non-lithium-medicated, non-suicidal individuals^12^.

^7^Li is an MR active nucleus with a spin of 3/2 and a relative sensitivity of 0.29 compared to 1 of protons. Due to this low MRI sensitivity in vivo lithium imaging has proven difficult. Moreover, lithium is a trace element with daily consumption estimates ranging from ~ 10 to 3000 µg per day^13,14^. So, the brain concentration in non-lithium-treated healthy humans is in the nano-to-micromolar range^13,15^, undetectable for currently available MRI methods. Since the therapeutic window of lithium is narrow^16^ and renal failure is a feared side effect, brain concentration cannot readily be increased. Exploring the physiological brain distribution of lithium in healthy humans in vivo is, therefore, almost impossible.

Several studies exploring the effect of lithium on the brain have used rodent models where lithium has been administered parenterally (mostly intraperitoneally) or orally (via food and drinking water) to achieve brain concentrations high enough for in vivo imaging. The significantly smaller brains, however, place high demands on spatial resolution. So far, the highest reported resolution of ^7^Li MRI in rodents is 2 × 2 × 4 mm^3^ and was acquired over 36 h using a turbo-spin echo sequence on ex vivo rat brains^17^. In contrast, in vivo ^7^Li MRI in rats was performed with a resolution of 4 × 4 × 7 mm^3^ (single slice)^18^, resulting in about 12 voxels per brain (volume of a rat brain: ~ 20 × 10 × 15 mm^3^). No in vivo ^7^Li MRI studies on mice have been reported so far.

**Figure 1:**
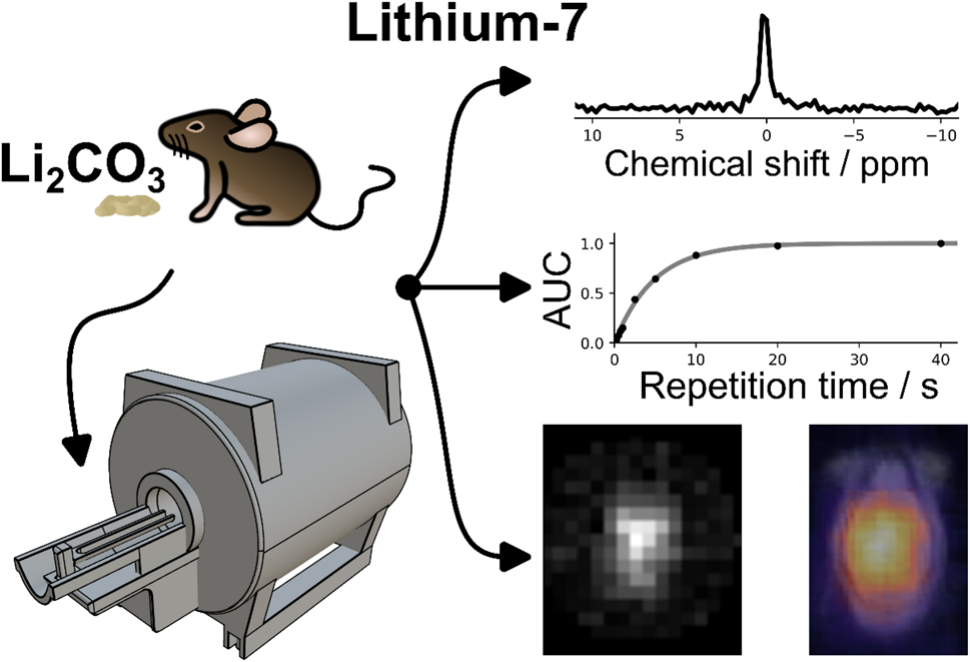
Graphical abstract of the study. In vivo ^7^Li MRI and MRS were acquired from 13 wild-type mice on a lithium-enriched diet (0.3% Li_2_CO_3_ w/w). Weekly ^7^Li MRS was performed to monitor the uptake of lithium. ^7^Li T1 relaxation time was estimated based on four mice. ^7^Li MRI was acquired using a SPIRAL sequence at a resolution of 2 × 2 × 3 mm^3^ with a 4-h acquisition time.

This study aims to find an MRI sequence suitable for in vivo ^7^Li MRI in mice. The protocol should provide a sufficient SNR in a measurement time still applicable in vivo. To be feasible in mice, we considered an SNR of more than five and an acquisition time of no longer than four hours. To that end, we first tested different MR sequences on lithium-containing phantoms. We explored the following four MR sequences: fast low-angle shot (FLASH), rapid acquisition with relaxation enhancement (RARE), balanced steady-state free precession (bSSFP), and SPIRAL. While FLASH and RARE sequences are commonly used in proton MRI, the efficient data acquisition of bSSFP and SPIRAL sequences make them promising candidates for in vivo ^7^Li MRI. In addition, bSSFP yields a high SNR, which depends on the ratio of transverse relaxation time (T2 relaxation) to the longitudinal relaxation time (T1 relaxation). The non-cartesian SPIRAL sequence uses a sinusoidal gradient switching, allowing a whole k-space acquisition in a single shot. Based on our results in phantoms, we then conducted an in vivo study in mice where we could show for the first time that in vivo ^7^Li MRI is feasible when using a SPIRAL sequence. A graphical summary of the study is shown in Fig. 1.

## Results:

### ^***7***^***Li MR spectroscopy (MRS)***

#### Lithium was reliably detectable in the brain already one week after treatment onset

**Figure 2:**
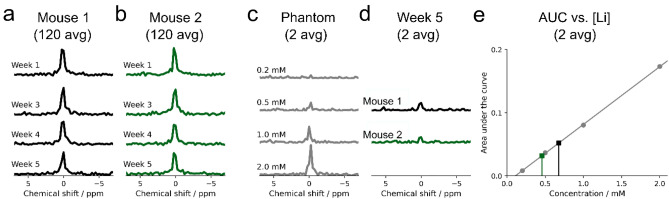
Lithium concentration in the brain. Follow-up localized ^7^Li MR spectra of two mice (**a**,**b**) were obtained weekly after the onset of oral lithium treatment. Using ^7^Li MR spectra of lithium-containing agarose phantoms (**c**) as a reference, the estimated brain concentration at week 5 was 0.68 mM for Mouse 1 and 0.46 mM for Mouse 2 (**d**). The linear relationship between the area under the curve and the known lithium concentrations of the phantom, together with the estimated brain concentration of Mouse 1 (black) and Mouse 2 (green) are shown in (**e**).

Feeding a lithium-enriched diet (0.3% w/w Li2CO3) resulted in a significant increase of brain lithium, observable at the first of the weekly acquired localized ^7^Li MR spectra, one week after the treatment started (Fig. 2a-b). The brain lithium did not change significantly over the following four weeks. To estimate the concentration, we compared the mice spectra with spectra acquired from agarose phantoms of different lithium concentrations (0.2 to 2.0 mM) using the same coil, identical acquisition parameters, and comparable object geometry and coil loading. In this way, we obtained an average brain lithium concentration of 0.6 mM (0.68 mM brain lithium for mouse 1 and 0.46 mM for mouse 2 at week five; Fig. 2c–e).

#### The average in vivo T1 relaxation time of ^7^Li at 9.4T was about 4.6 s

**Figure 3:**
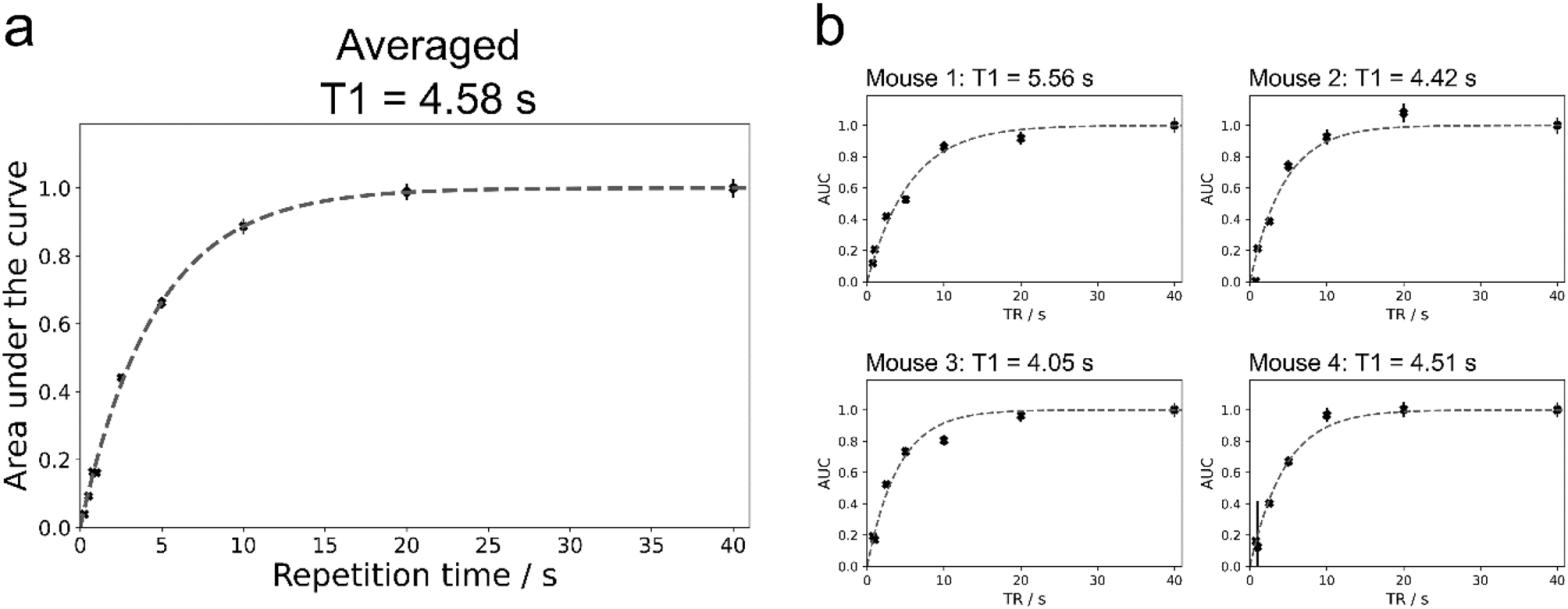
T1 relaxation time of brain lithium in vivo at 9.4T. (**a**) The averaged area under the curve (AUC) obtained from the average ^7^Li MR spectrum of four mice at different repetition times reveals a T1 relaxation time of 4.58 s. The individual results of each mouse are shown in (**b**). AUC values are shown with error bars indicating the standard deviation of the AUC.

We acquired non-localized ^7^Li spectra from the mouse head with varying repetition times (Fig. 3). The exponential fitting to estimate T1 relaxation time was approached in two different ways, which led to comparable results: first, we used the area under the curve (AUC) of the averaged spectrum of four mice (T1 = 4.58 ± 0.13 s, Fig. 3a), and second, the signal curve of each mouse was processed individually, resulting in a T1 relaxation time of 4.64 ± 0.56 s (Fig. 3b).

#### Ex vivo ^7^Li MRS revealed significant lithium wash-out within 27 h after PFA fixation

Taking advantage of the possibility of longer acquisition time, we performed *post-mortem* lithium measurements of the isolated brain. Following the recommendation of Stout et al.^17^, we excised the brain directly after transcardial perfusion fixation and placed it in 5 ml PFA to limit lithium efflux. However, ^7^Li MRS acquired in intervals over 74 h, showed a substantial signal reduction, reaching the detection limit of lithium in the brain after about 27 h (Fig. S1). Logistic curve fitting revealed a half-maximum at 12.7 h and a decay rate of 0.21. A steady state (95% decay) was reached after 27 h.

### ^7^Li MR imaging

#### Sensitivity profile of the ^7^Li radiofrequency coil

**Figure 4:**
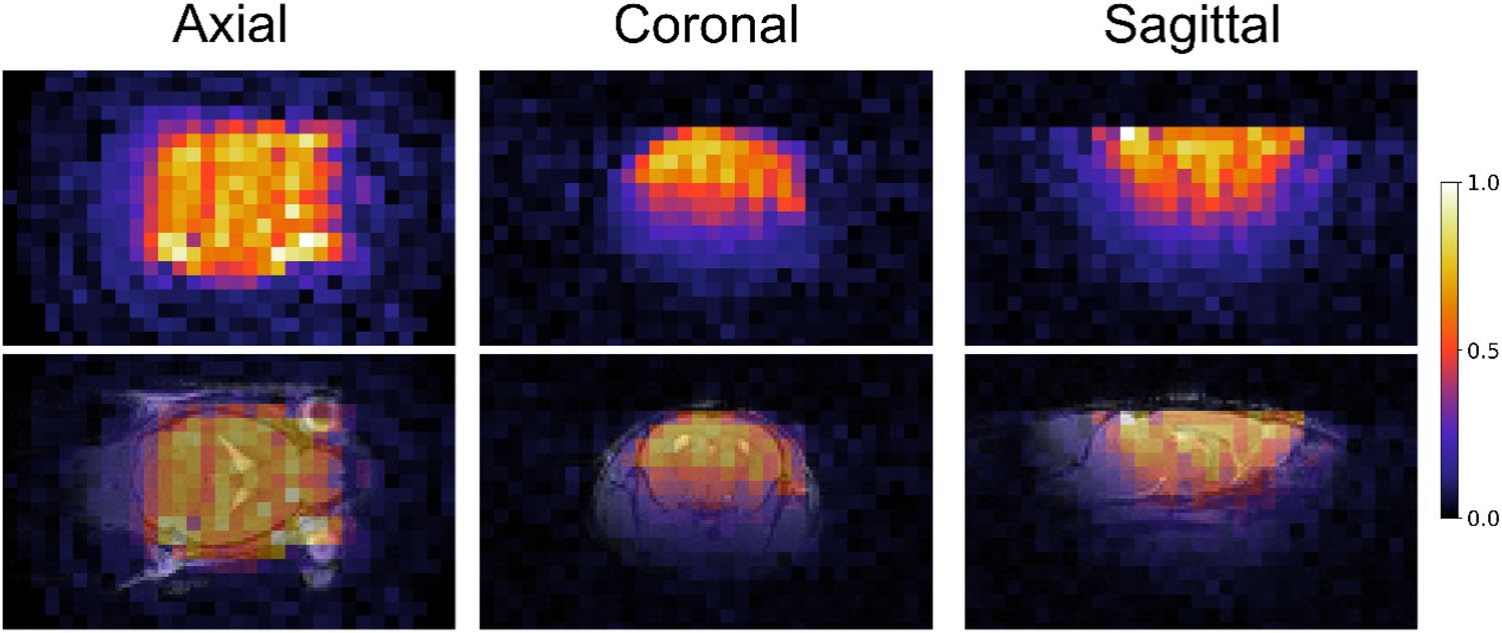
Sensitivity profile of the ^7^Li coil. A SPIRAL ^7^Li MRI was acquired on an agarose phantom containing 10 mM LiCl (top row). The bottom row shows the ^7^Li image of the phantom overlaid on a ^1^H image of a mouse head to illustrate the coverage of the brain.

The coil sensitivity profile of the single-loop transmit-receive coil (inner diameter 17 mm) was acquired on an agarose phantom (10 mM LiCl) and overlaid on a T2-weighted anatomical image of a mouse brain, Fig. 4. The profile covered almost completely the rostrocaudal and left–right dimensions of the brain. In the anteroposterior direction, the surface coil showed the typical sensitivity loss with increasing distance. However, the predominant brain parts were within the coil’s best sensitivity region.

#### SPIRAL ^7^Li MRI had the highest SNR per unit acquisition time

**Figure 5:**
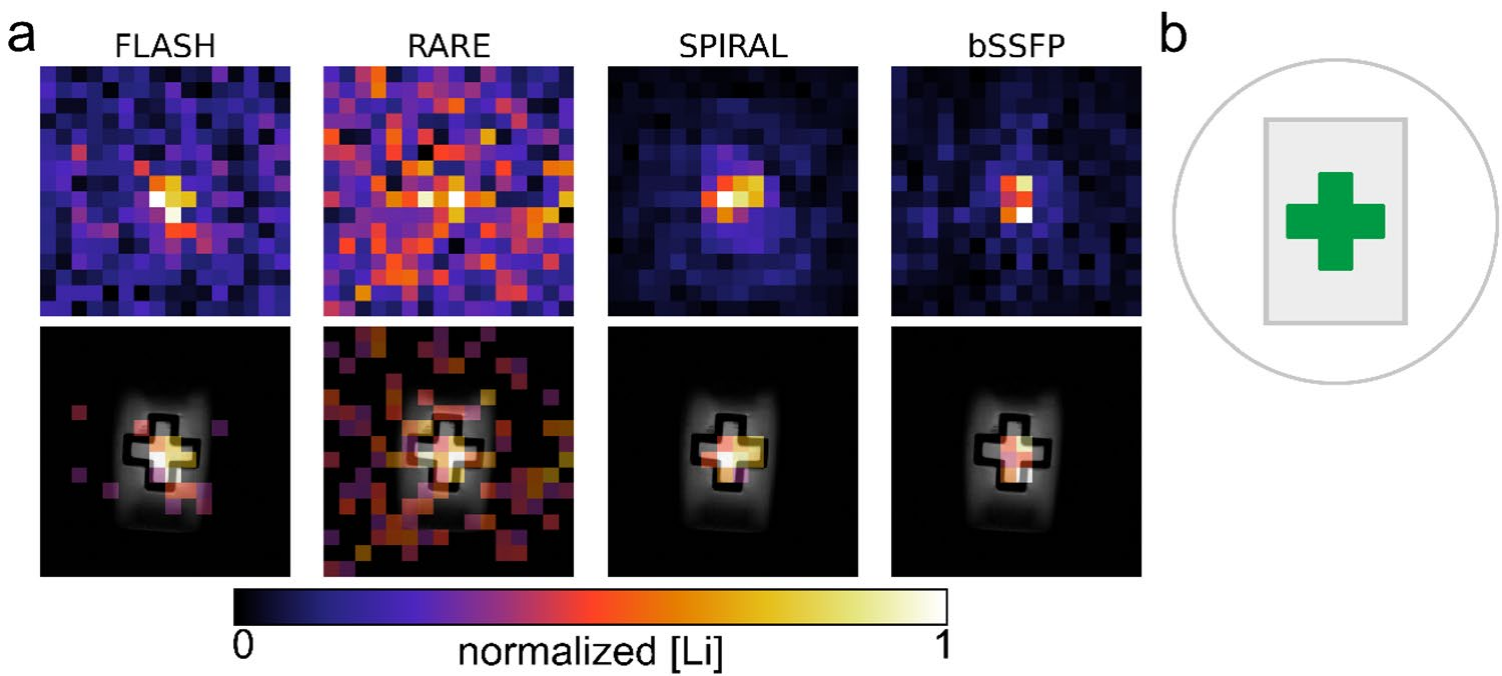
^7^Li MR images acquired within 4 h using either FLASH, RARE, SPIRAL or a bSSFP squence. The top row shows the phantom’s ^7^Li images, and the bottom shows them thresholded and overlaid on a ^1^H reference image (**a**). A sketch of the phantom (**b**) illustrates the circular field of view of the SPIRAL sequence. The green plus sign contained 1.0 mM LiCl. The threshold of the ^7^Li images was set to one-third the maximum intensity of the ^7^Li image. The FLASH and RARE sequences performed significantly worse than the SPIRAL and bSSFP sequences. However, the signal from the FLASH images still originated primarily from the lithium-containing plus-shaped center as also shown for SPIRAL and bSSFP. The highest SNR was obtained with the SPIRAL sequence (SNR = 8.2) followed by bSSFP (SNR = 6.8).

A protocol feasible for in vivo ^7^Li MRI in mice should be able to detect lithium at a therapeutic relevant brain concentration (~ 1 mM), a spatial resolution of at least 2 × 2 × 3 mm^3^, and within a measurement time, being still achievable in vivo. We compared the SNR of four different sequences (SPIRAL, RARE, bSSFP, and FLASH) using a structured phantom that contained 1 mM LiCl in agarose in its center, Fig. 5b. We acquired two coronal slices to obtain a field of view that covers the entire mouse brain. Under these conditions, the SPIRAL sequence achieved the highest SNR (8.2), followed by bSSFP (SNR = 6.8), FLASH (SNR = 3.2), and in the last place, the RARE sequence (SNR = 1.8) (Fig. 5a).

#### SPIRAL ^7^Li MRI provided the best SNR in vivo

**Figure 6:**
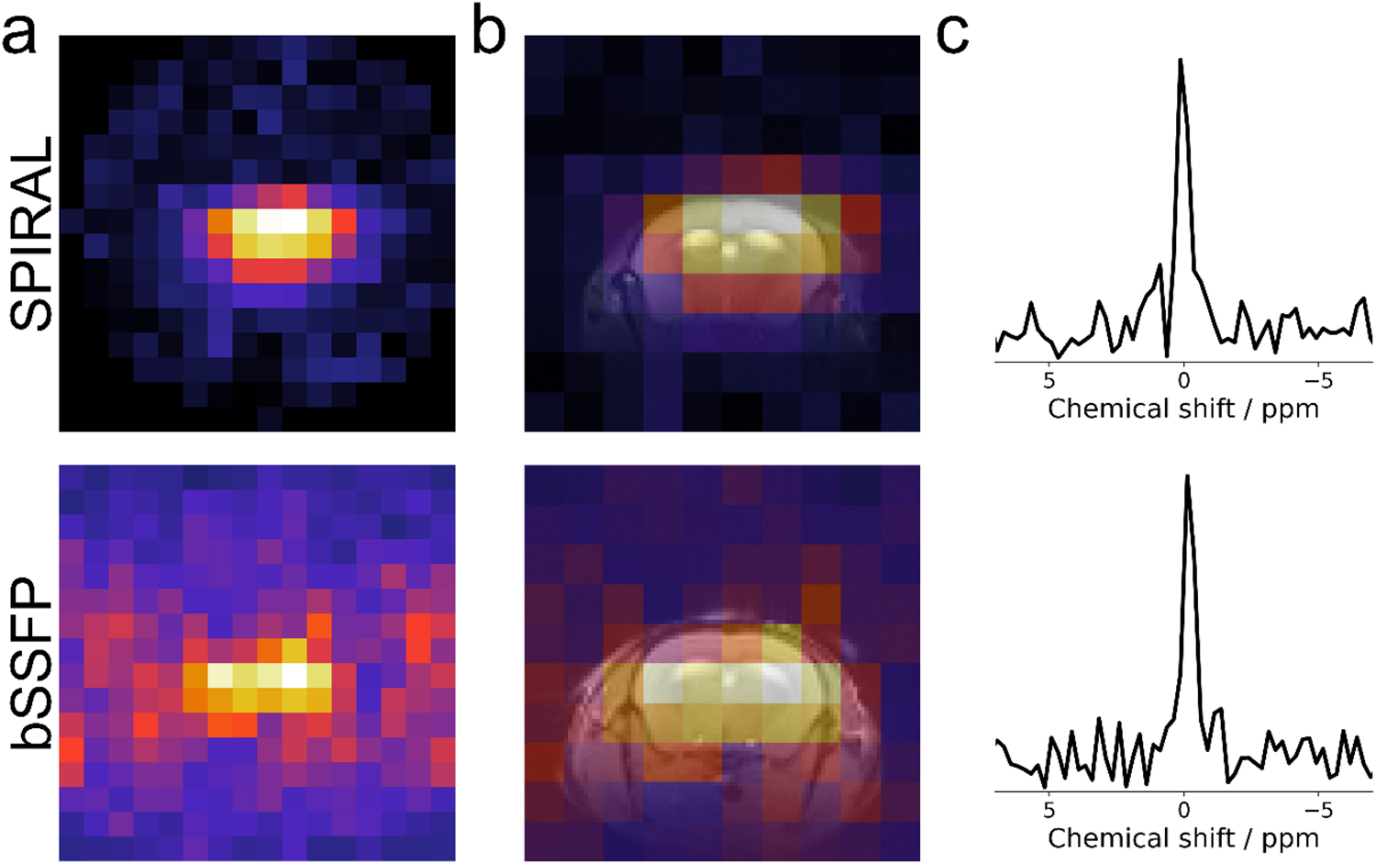
SPIRAL ^7^Li MRI had higher SNR than bSSFP ^7^Li MRI in vivo. The raw (**a**) and overlaid (**b**) ^7^Li images show lithium signals originating mainly from the brain. The ^7^Li image acquired with a SPIRAL sequence had higher SNR than the bSSFP (6.7 vs. 2.3) despite the same acquisition time and comparable brain lithium concentration as shown by the respective localized ^7^Li spectra (**c**).

Next, we applied the two sequences with the highest in vitro SNR, SPIRAL and bSSFP, each on a lithium-treated mouse in vivo. Although the two mice did not show differences in their brain lithium level, as revealed by ^7^Li MRS in the same session (Fig. 6c), bSSFP exhibited a noticeably lower SNR than SPIRAL (2.3 vs. 6.7, Fig. 6a,b).

#### In vivo ^7^Li SPIRAL MRI is feasible at a resolution of 2 × 2 × 3 mm^3^.

**Figure 7:**
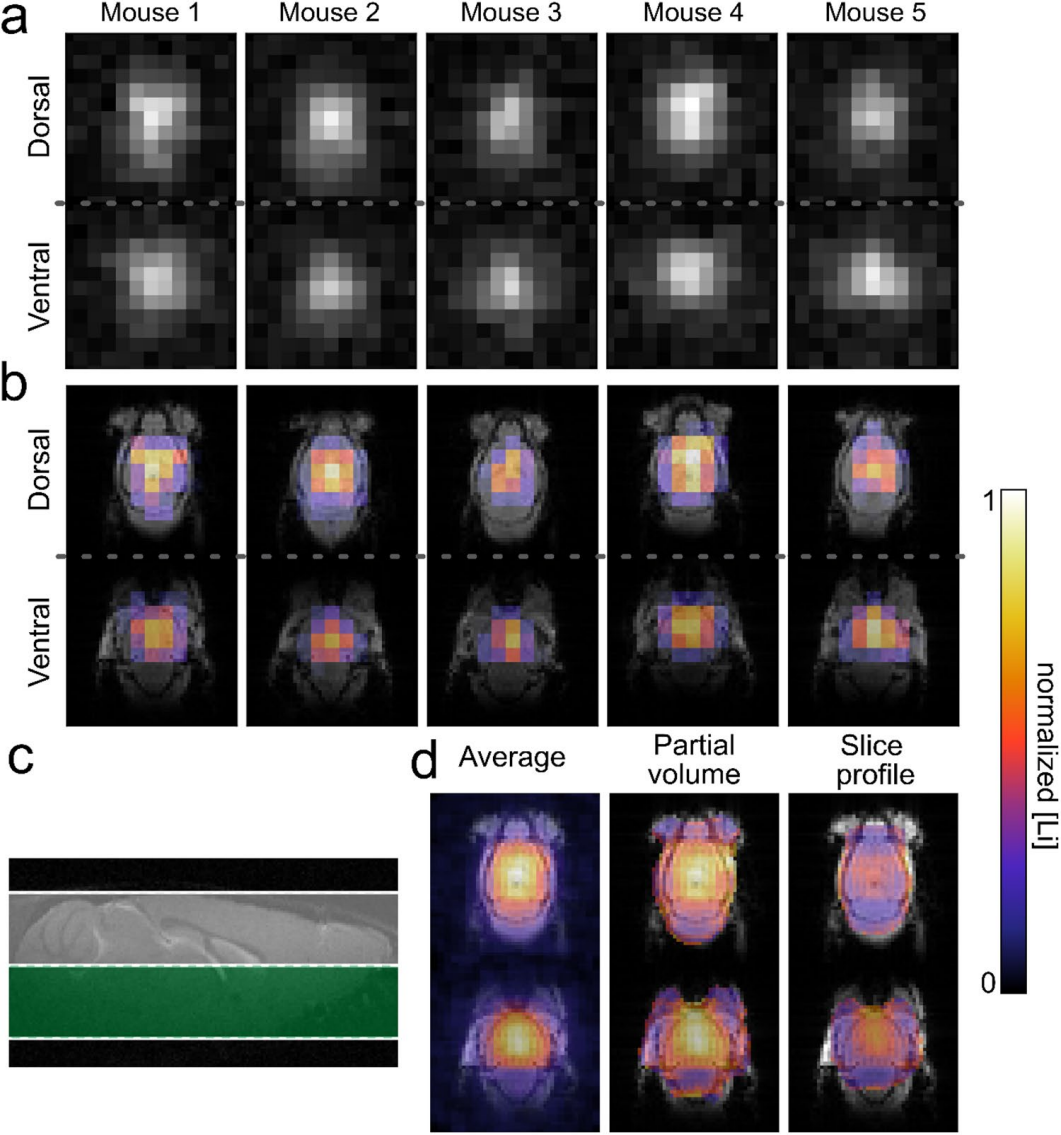
In vivo ^7^Li MRI of lithium-fed, wild-type mice. ^7^Li MRI was acquired in five mice using a 4-h SPIRAL sequence (**a**,**b**). The position of the two coronal slices is visualized in **c**. The brains were segmented, and the ^7^Li images were overlaid on ^1^H reference images (**b**). The average ^7^Li image (**d**, left) showed the highest lithium signal in the brain’s center. After partial volume correction (**d**, center), we also observed a lithium signal at the brain’s edges. The ^7^Li image, additionally corrected for the signal intensity profile of the coil (**d** right), shows the highest lithium signal in the main parts of the cerebrum and less in the olfactory bulb and cerebellum.

**Figure 8:**
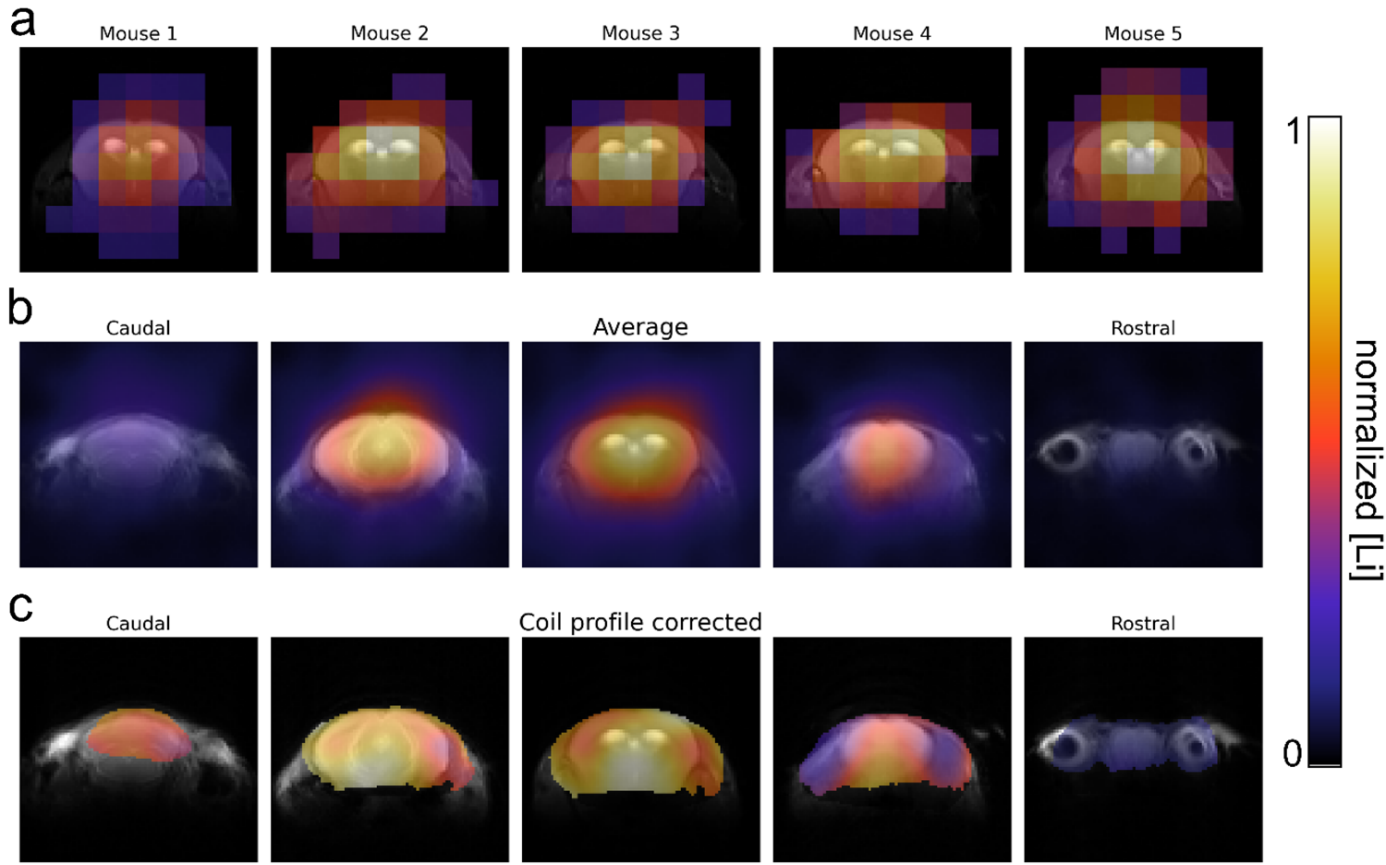
Axial ^7^Li MRI showed high lithium signal in main parts of the cerebrum and low signal in the cerebellum and olfactory bulb. In vivo ^7^Li MRI of five mice, performed in the axial orientation (**a**), showed a clear lithium signal originating from the brain. The averaged ^7^Li image of the five mice revealed the highest lithium concentration in the brain center and only shallow signals in the cerebellum and olfactory bulb (**b**). These findings remained after correcting for the coil profile (**c**).

Having SPIRAL identified as, in our hands, the most suited sequence, we acquired in vivo ^7^Li SPIRAL MRI in 10 mice after 4–5 weeks of lithium treatment. In one-half of the mice, the images were obtained in two coronal slices (visualized in Fig. 7c); for the other half, an axial orientation was chosen. Lithium was detectable in the brains of all mice (Figs. 7a,b and 8a). We calculated the average ^7^Li image for the two slice orientations separately, using the centroid alignment of the respective five mouse brains. The group average ^7^Li images showed a prominent lithium signal in the center of the brain, including the lateral ventricles and deep grey matter nuclei (Figs. 7d and 8b). Interestingly, the olfactory bulb exhibited almost no lithium signal. This fact remained when correcting for the sensitivity profile of the coil and voxel proportions exceeding the head of the mouse (Figs. 7d and 8c). Compared to the cerebrum, the cerebellum showed a notably lower lithium signal.

## Discussion

^7^Li MRS and MRI can provide valuable information about the pharmacokinetics and distribution of lithium in the brain. Combined with the vast number of available genetically modified mouse lines, these in vivo techniques may further help to unravel its mood regulation mechanisms. To enable ^7^Li MRI in the tiny mouse brain, we tested four MR sequences, namely FLASH, RARE, bSSFP, and SPIRAL, on lithium-containing phantoms, followed by in vivo experiments in mice using the two best sequences bSSFP and SPIRAL. We could show for the first time that in vivo ^7^Li MRI in mice is feasible, although with a limited spatial resolution and SNR. In this context, the fast non-Cartesian sampling of the SPIRAL sequence significantly improved the SNR and image quality.

Feeding mice with a lithium-enriched diet led to a detectable lithium concentration in the brain one week after the treatment started. The estimated brain concentration of 0.6 mM aligns with a previous *post-mortem* study reporting a brain concentration of about 0.8 mM after lithium treatment using time-of-flight secondary ion mass spectrometry on juvenile mice^19^. Assuming a brain-to-blood ratio of roughly 1.2 in C57BL mice^20^, the lithium treatment used in this study corresponds to a lithium serum concentration within the upper therapeutic window of 0.4–1.2 mM in patients^19,21^.

Our estimated T1 relaxation of brain lithium at 9.4T (4.58 s) is comparable to previous reports in humans at 7T (3.95 s) and 4T (4.12 s) and also aligns with measurements obtained in rats at 4.7T (2.5–5.1 s)^10,22,23^. This T1 relaxation time was considered when comparing the four MR sequences. In vitro and in vivo, SPIRAL provided the highest SNR per unit acquisition time. Its single-shot, center-out encoding allowed for a short echo time (TE), low bandwidth per pixel, and efficient k-space sampling. Using the SPIRAL sequence, we could achieve a spatial resolution of 2 × 2 × 3 mm^3^ within an acquisition time of four hours. Given a mouse brain size of about ~ 10 × 6 × 12 mm^3^ this resolution means approximately 15 voxels per coronal brain slice.

We corrected the signal intensity for the tissue percentage in each voxel to compensate for partial volume effects at the brain edges caused by the relatively thick coronal slices. Another limitation was the sensitivity profile of the circular surface coil. We took this into consideration by weighting the obtained images with the sensitivity profile of the receiver coil acquired on a homogeneous lithium-containing phantom resembling the size and shape of a mouse head.

The ^7^Li images corrected this way indicated a non-homogeneous distribution of lithium in the healthy mouse brain. The highest concentration was found in the brain center, including large parts of the cortex, lateral ventricles, and basal ganglia. We observed significantly lower concentrations in the cerebellum and olfactory bulb. The former contrasts a *post-mortem* study in juvenile mice^19^, where the authors reported an accumulation of lithium in neurogenic regions such as the hippocampus and the olfactory bulb. Although low levels remain in the hippocampus and olfactory bulb of the adult brain, neurogenesis peaks during early development. It is hitherto undescribed whether juvenile and adult mice have different lithium distributions. However, our observation indicates that the neurogenesis level may be responsible for potential age-related differences. Moreover, ex vivo rat ^7^Li MRI found higher lithium levels in the cortex and lower in the brainstem^17^. In addition, the rodent cerebellum, especially in white matter, has been shown to have lower lithium concentrations, per our findings^17,24^.

In humans, it has been shown that lithium is found in higher concentrations in the brainstem and structures of the limbic system^10^. Our coil does not cover the brainstem and as such we cannot compare these findings; however, we do observe increased lithium in the basal ganglia and at the center of the brain comparable to human studies^10^.

*Post-mortem* ^7^Li MRI theoretically reveals the possibility of spatially higher resolved images. However, in contrast to an ex vivo rat study^17^, we observed a significant wash-out of lithium from the paraformaldehyde (PFA) fixed brain into the solution. In the future, other fixation techniques need to be found to profit from the possible prolonged measurement time in *post-mortem* studies.

In conclusion, to our knowledge, we have acquired the first ^7^Li MRS and ^7^Li MRI of a mouse brain in vivo. ^7^Li MRI of mice was feasible within 4 h at a resolution of 2 × 2 × 3 mm^3^. Although restricted in anatomical precision, we found indications that the in vivo distribution of lithium in the brain may not be homogeneous. SPIRAL ^7^Li MRI provides a new tool for studying lithium treatment and response in mice. It may link regional lithium concentrations and structural or metabolic changes. While still in its infancy, murine in vivo ^7^Li MRI may help to increase our understanding of lithium’s mode of action.

## Materials and methods

### Animals

13 adult C57BL/6N mice (8 female, 5 male) were enrolled in the study. The study was approved by the local ethics committee (Animal Welfare Service, Lower Saxony State Office for Consumer Protection and Food Safety, license-number 33.19-42502-04-20/3365). The study design complies with the ARRIVE guidelines and experiments were performed in accordance with relevant guidelines and regulations (Directive 2010/63/EU, European Parliament on the protection of animals used for scientific purposes). The mice were kept on a 12-h light–dark cycle. Food (ssniff Spezialdiäten GmbH) and water were provided ad libitum. Water consumption was monitored daily. A saline solution was additionally provided when water consumption increased by 300% (B. Braun Medical Inc., Bethlehem, Pennsylvania, USA). In analogy to the treatment commonly used in humans, all mice received a lithium-enriched diet containing 0.3% Li_2_CO_3_ (w/w).

For each MR session, the mice were initially anaesthetized with ketamine (MEDISTAR, Serumwerk Bernburg Tiergesundheit GmbH, Bernburg, Germany) and medetomidine (Dorbene Vet, Zoetis Inc., Parsippany, New Jersey, USA), intubated and subsequently artificially ventilated (animal respirator, advanced 4601-2, TSE Systems GmbH, Bad Homburg, Germany). Anesthesia was maintained by 0.5–1.5% isoflurane (Isofluran CP, CP-Pharma Handelsgesellschaft mbH, Burgdorf, Germany) in oxygen-enriched air. Breathing rate and rectal temperature were continuously monitored. At the end of the measurement, medetomidine was antagonized by atipamezole (Atipzole, Provident Pharmaceuticals, Prodivet Pharmaceuticals sa/nv, Eynatten, Belgium). After the final MR session, the mice were transcardially perfused with phosphate-buffered saline solution and 4% PFA (Carl Roth GmbH, Karlsruhe, Germany) and the brains were collected for *post-mortem* analyses.

### MR system

MR data was acquired on a 9.4T MRI system (BioSpec, 30 cm horizontal bore, BGA12 gradient system, ParaVision 6.0.1; Bruker BioSpin MRI GmbH, Ettlingen, Germany). A dual-tuned (^1^H/^7^Li) transmit-receive surface coil (RAPID Biomedical GmbH, Rimpar, Germany) was used for both ^1^H and ^7^Li measurements. The ^7^Li channel had a single-loop design with a diameter of 17 mm. The optimal reference power was determined manually by acquiring multiple non-localized ^7^Li spectra with increasing reference power and a long repetition time (TR = 40 s) to eliminate T1 relaxation effects.

### Chemicals

Agarose and LiCl used for phantom experiments were acquired from Carl Roth (Carl Roth GmbH + Co. KG, Karlsruhe, Germany).

### Magnetic resonance spectroscopy

Axial and sagittal T2-weighted ^1^H images were acquired to help position the spectroscopy voxel (2D RARE sequence, TR = 2800 ms, TE = 33 ms, RARE-factor = 8, 0.1 × 0.1 mm^2^ resolution, 19.2 × 19.2 mm^2^ field of view, 0.5 mm slice thickness, 0.3 mm slice gap, 24 slices, 2 averages, and 2:14 min acquisition time). Localized ^7^Li MR spectra were obtained using an image-selected in vivo spectroscopy (ISIS) sequence^25^ with a spectral width of 10 kHz, a voxel size of 6 × 5 × 8 mm^3^, and 256 data points. In vivo ^7^Li MR spectra (TR = 2.5 ms, number of averages (NA) 120 resulting in a total acquisition time of 40 min) were acquired weekly from two mice starting at age 146 days, one week after the lithium treatment onset, and continuing for the following four weeks to monitor the brain lithium uptake. No data could be obtained in week two due to technical problems with the MR system. Subsequent to the in vivo experiments, one mouse brain was analyzed in a 4% PFA solution using the same ISIS protocol to investigate the ex vivo lithium wash-out. The spectra were acquired over 74 h with interleaved ^1^H reference images to ensure an unchanged voxel position.

To estimate the in vivo lithium concentration in the brain, ^7^Li MR spectra from phantoms with a comparable size to mouse heads (2 ml, Sarstedt AG & Co. KG, Nümbrecht, Germany) containing lithium concentrations in the range of 0.2–2 mM ([LiCl] in agarose given in % weight per volume water: 0.2 mM/3.00%, 0.5 mM/2.99%, 1 mM/2.98%, 2 mM/2.95%) were acquired as a reference and compared with those spectra additionally obtained from the mice in week five using identical acquisition parameters (TR = 40 s, NA = 2, total acquisition time = 10.7 min). The long TR was chosen to minimize the effect of the different T1 relaxation times between the phantom and the brain.

In addition, we acquired non-localized spectra (spectral width 16,026 Hz, 256 data points, NA = 10) in four mice to estimate the T1 relaxation time of ^7^Li in the brain. The spectra were obtained with nine different TRs (0.25 s to 40 s) from longest to shortest TR with 80 s of dummy scans to ensure a steady state during the acquisition.

### Magnetic resonance imaging

To find a suitable sequence for in vivo ^7^Li MRI in mice, four different MR sequences, i.e. 2D FLASH, 2D RARE, 2D bSSFP, and single-shot 2D SPIRAL, were tested on a phantom containing 1 mM aqueous LiCl solution in its cross-shaped center surrounded by lithium-free agarose (2.94%). Two coronally oriented slices were obtained with a resolution of 2 × 2 × 3 mm^3^. The total acquisition time was 4 h for each of the four sequences. The respective MR parameters are shown in Table 1. For FLASH, RARE, and SPIRAL, the flip angle was calculated using the Ernst angle formula and an estimated T1 of 11 s for ^7^Li in an aqueous solution^26^. For SPIRAL ^7^Li MRI, a bandwidth of 7500 Hz was found to be optimal to balance the effects of sampling time and T2* decay, yielding both good SNR and spatial acuity. ^1^H reference images were acquired with a FLASH sequence, 125 µm isotropic in-plane resolution and 3 mm slice thickness.

**Table 1:** MR parameters used for sequence comparison on the lithium-containing phantom shown in Fig. 5.

	RARE	FLASH	bSSFP	SPIRAL
TR/ms	2500	2500	5	2500
TE/ms	6.73	1.52	2.5	1.58
Flip angle	36.1°	36.1°	20°	36.1°
N_avg_	60	15	35,018	5760
Matrix size	32 × 32	32 × 32	16 × 16	16 × 16
Slices	2	2	2	2
Voxel size/mm^3^	2 × 2 × 3	2 × 2 × 3	2 × 2 × 3	2 × 2 × 3
FOV/mm^2^	64 × 64	64 × 64	32 × 32	32 × 32
t_acq_/hr	4	4	4	4
Sequence-specific parameters	RARE-factor: 4			Bandwidth: 7500 Hz

The signal intensity profile of the ^7^Li coil was measured on a phantom with a volume of 5 ml (ClearLine CLEAR-LOCK, Kisker Biotech GmbH & Co. KG, Steinfurt, Germany) containing 10 mM aqueous LiCl solution. To cover a sufficiently large volume, 20 slices with a spatial resolution of 1 × 1 × 1 mm^3^ (matrix size 32 × 32) were acquired within 18 h (NA = 25,920) using a SPIRAL sequence with the same TE, TR and flip angle used in vivo.

Eleven of the thirteen mice underwent ^1^H and ^7^Li MRI. Axial and coronal ^1^H reference images were acquired using either a 2D RARE sequence (TR = 2800 ms, TE = 33 ms, 0.2 mm isotropic in-plane resolution, 32 × 32 mm^2^ field of view, 1 mm slice thickness, 21 slices, 5 averages, and 4:40 min acquisition time, Figs. 6 and 8), a 2D FLASH sequence (TR = 100 ms, TE = 2 ms, 0.5 × 0.5 mm^2^ resolution, 32 × 32 mm^2^ field of view, 0.5 mm slice thickness, 12 slices, 2 averages, 6 dummy scans, and 2:34 min acquisition time, Fig. 7) or 3D SSFP sequence (TR = 5 ms, TE = 2.5 ms, 0.25 × 0.25 × 0.25 mm^3^ resolution, 36 × 36 × 36 mm^3^ field of view, 2 averages, and 4:47 min acquisition time, Fig. 6—bottom). For in vivo sequence comparisons, axial-oriented ^7^Li MR images was obtained using a SPIRAL (five mice) or a bSSFP (one mouse) sequence. The respective MR parameters are summarized in Table 2. Finally, using ^7^Li SPIRAL MRI seven axial-oriented or two coronal-oriented slices were acquired from five mice each.

**Table 2:** MR parameters used for in vivo sequence comparison – Fig. 6.

	SPIRAL	bSSFP
TR/ms	2500	5
TE/ms	1.58	2.5
Flip angle	53.3°	20°
N_avg_	5760	4800
Matrix size	16 × 16	24 × 24 × 24
Slices	7	3D sequence
Voxel size/mm^3^	2 × 2 × 3	1.5 × 1.5 × 1.5
t_acq_/h	4	4

### Data processing and analysis

MRI and MRS data were processed and analyzed using MATLAB (The MathWorks, Inc., Natick, Massachusetts, USA) and Python (version 3.7.9, Python Software Foundation). The final figures were assembled in Inkscape (The Inkscape Project).

^7^Li magnitude spectra were subtracted by the mean of the noise, defined as the mean of the 100 data points furthest from the ^7^Li resonance. The AUC of each spectrum was calculated using three-parameter Lorentzian fitting (AUC, chemical shift, and full-width at half maximum [FWHM]) with the help of *curve_fit* from the *SciPy* toolbox *optimize*. The following boundary conditions were applied: chemical shift = [− 5, 5 ppm], FWHM = [0.2, 1.0 ppm] for the localized spectra and FWHM = [0.2,1.5 ppm] for non-localized spectra, and AUC = [0, 100]. The fit parameters for the weekly ^7^Li spectra are given in Table S1.

The in vivo concentration of lithium in the brain was estimated by comparing the AUC of the in vivo ^7^Li spectra with those obtained from phantoms of known ^7^Li concentrations, assuming a linear relationship between AUC and ^7^Li concentration.

To estimate the in vivo T1 relaxation time of ^7^Li, we averaged the spectra across the four mice. All AUCs were normalized to the AUC for the longest TR (40 s). The normalized data was fitted to the T1 relaxation equation (Eq. 1, *S*—relative signal intensity). In addition, similar fits were performed for each mouse spectrum individually. Here spectra with a TR < 1 s had to be excluded due to poor SNR. The fit parameters for the estimation of the T1 relaxation time are shown in Table S2 (visualized in Fig. S2).

1 $$S=1-{\text{exp}}\left(-\frac{TR}{T1}\right)$$

Moreover, to quantify the ex vivo lithium wash-out, the normalized AUC as a function of time was fitted with a logistic curve and the following parameters: amplitude, inflexion point, and steady-state signal intensity.

To compare the four MR sequences explored for ^7^Li MRI, the field of view of the FLASH and RARE images was reduced to 32 × 32 mm^2^. The SNR of each image was measured as the mean intensity of the signal inside the region of interest (ROI), (in green Fig. 5b), divided by the mean intensity of the noise. The ROI was defined based on ^1^H reference image, down-sampled to an in-plane resolution of 2 × 2 mm^2^ and binarized.

To take the signal intensity profile of a surface coil into account the ^7^Li image obtained from a homogeneous phantom was down-sampled to a resolution of 2 × 2 × 3 mm^3^, smoothed with a Gaussian filter (σ = 0.5), and normalized between 0 and 1. This mask was then used to weight the pixels of the in vivo ^7^Li MR images, respectively. In addition, ^7^Li images were corrected for partial volume effects. The percentage of tissue in each voxel of the ^7^Li image was calculated from the binarized ^1^H reference images (tissue vs no tissue). To minimize over-correction, we excluded voxels with low tissue content (Otsu threshold; tissue content > 38%).

The ^7^Li images were overlayed on a ^1^H reference image for better visualization. To that end, the ^7^Li images were resized to the resolution of the ^1^H reference images using nearest neighbour interpolation. The threshold for the ^7^Li signal was computed by maximizing the inter-class variance using either MATLAB or the *scikit-image* library in Python, both based on Otsu’s method^27^. Finally, the ^7^Li images of five mice were first corrected for partial volume (coronal images only) and the coil profile and then averaged using centroid alignment.

### Supplementary Information

Supplementary Information.

## Data Availability

The datasets generated during and analysed during the current study are available from the corresponding author on reasonable request.

## Abbreviations

MR: Magnetic resonance
MRI: Magnetic resonance imaging
MRS: Magnetic resonance spectroscopy
SNR: Signal-to-noise ratio
AUC: Area under the curve
ISIS: Image-selected in vivo spectroscopy
RARE: Rapid acquisition with relaxation enhancement
FLASH: Fast low-angle shot
bSSFP: Balanced steady-state free precession
T2 relaxation: Transverse relaxation
T1 relaxation: Longitudinal relaxation
PFA: Paraformaldehyde
ROI: Region of interest
TR: Repetition time
TE: Echo time

## References

[1] Alda M (2015). Lithium in the treatment of bipolar disorder: Pharmacology and pharmacogenetics. Mol Psychiatry.

[2] Nonaka S, Hough CJ, Chuang D-M (1998). Chronic lithium treatment robustly protects neurons in the central nervous system against excitotoxicity by inhibiting N-methyl-d-aspartate receptor-mediated calcium influx. Proc. Natl. Acad. Sci. USA.

[3] Harwood AJ (2005). Lithium and bipolar mood disorder: The inositol-depletion hypothesis revisited. Mol. Psychiatry.

[4] Kolczynska K, Loza-Valdes A, Hawro I, Sumara G (2020). Diacylglycerol-evoked activation of PKC and PKD isoforms in regulation of glucose and lipid metabolism: A review. Lipids Health Dis..

[5] Goode N, Hughes K, Woodgett JR, Parker PJ (1992). Differential regulation of glycogen synthase kinase-3 beta by protein kinase C isotypes. J. Biol. Chem..

[6] Böer U (2007). Enhancement by lithium of cAMP-induced CRE/CREB-directed gene transcription conferred by TORC on the CREB basic leucine zipper domain. Biochem. J..

[7] Quiroz JA, Machado-Vieira R, Zarate CA, Manji HK (2010). Novel insights into lithium’s mechanism of action: Neurotrophic and neuroprotective effects. Neuropsychobiology.

[8] Alda M (2017). Who are excellent lithium responders and why do they matter?. World Psychiatry.

[9] Smith FE (2018). 3D 7Li magnetic resonance imaging of brain lithium distribution in bipolar disorder. Mol. Psychiatry.

[10] Stout J (2020). Accumulation of lithium in the hippocampus of patients with bipolar disorder: A lithium-7 magnetic resonance imaging study at 7 Tesla. Biol. Psychiatry.

[11] Lee J-H (2012). 4-T 7Li 3D MR spectroscopy imaging in the brains of bipolar disorder subjects. Magn. Reson. Med..

[12] Schoepfer J (2021). Position sensitive measurement of trace lithium in the brain with NIK (neutron-induced coincidence method) in suicide. Sci. Rep..

[13] Schrauzer GN (2002). Lithium: Occurrence, dietary intakes, nutritional essentiality. J. Am. Coll. Nutr..

[14] Filippini T (2020). Dietary estimated intake of trace elements: Risk assessment in an Italian population. Expo. Health.

[15] Liaugaudaite V, Mickuviene N, Raskauskiene N, Naginiene R, Sher L (2017). Lithium levels in the public drinking water supply and risk of suicide: A pilot study. J. Trace Elem. Med. Biol..

[16] Nolen WA (2019). What is the optimal serum level for lithium in the maintenance treatment of bipolar disorder? A systematic review and recommendations from the ISBD/IGSLI Task Force on treatment with lithium. Bipolar Disord..

[17] Stout J (2017). Investigation of lithium distribution in the rat brain ex vivo using lithium-7 magnetic resonance spectroscopy and imaging at 17.2 T. NMR Biomed..

[18] Ramaprasad S, Newton JEO, Cardwell D, Fowler AH, Komoroski RA (1992). In vivo 7Li NMR imaging and localized spectroscopy of rat brain. Magn. Reson. Med..

[19] Zanni G (2017). Lithium accumulates in neurogenic brain regions as revealed by high resolution ion imaging. Sci. Rep..

[20] Hamburger-Bar R, Robert M, Newman M, Belmaker RH (1986). Interstrain correlation between behavioural effects of lithium and effects on cortical cyclic AMP. Pharmacol. Biochem. Behav..

[21] O’Donnell KC, Gould TD (2007). The behavioral actions of lithium in rodent models: Leads to develop novel therapeutics. Neurosci. Biobehav. Rev..

[22] Forester BP (2009). Brain lithium levels and effects on cognition and mood in geriatric bipolar disorder: A lithium-7 magnetic resonance spectroscopy study. Am. J. Geriatr. Psychiatry.

[23] Komoroski RA, Pearce JM (2004). Localized 7Li MR spectroscopy and spin relaxation in rat brain in vivo. Magn. Reson. Med..

[24] Heurteaux C (1991). Lithium transport in the mouse brain. Brain Res..

[25] Ordidge RJ, Connelly A, Lohman JAB (1986). Image-selected in Vivo spectroscopy (ISIS). A new technique for spatially selective nmr spectroscopy. J. Magn. Reson..

[26] Ernst RR, Anderson WA (1966). Application of Fourier transform spectroscopy to magnetic resonance. Rev. Sci. Instrum..

[27] Otsu N (1979). A threshold selection method from gray-level histograms. IEEE Trans. Syst. Man Cybern..

