# Supplementary material for: SPIRAL MRI for in vivo lithium-7 imaging: a feasibility study in mice after oral lithium treatment
Source: Sci Rep. 2024 Jan 5;14:681. doi: 10.1038/s41598-023-50841-7 (PMC10770043; doi:10.1038/s41598-023-50841-7)
Supplement: Supplementary file 1 — Supplementary Information. [file 41598_2023_50841_MOESM1_ESM.docx]

# Title: SPIRAL MRI for in vivo Lithium-7 Imaging – A feasibility study in mice after oral lithium treatment

## Authors and affiliations:

Tor Rasmus Memhave
Amir Moussavi, Dr.
Susann Boretius, Prof. Dr.

# Supplementary Information:

## Measuring Lithium wash-out:

**Fig. S1:** **Lithium wash-out after PFA fixation.** (a) Localized (green box in b) ^7^Li spectra of the isolated brain in PFA solution were continuously acquired over 74 hours. (b) The normalized area under the curve illustrates the lithium wash-out with an inflexion point at 12.7 hours and reaching a steady state after ~27 hours.


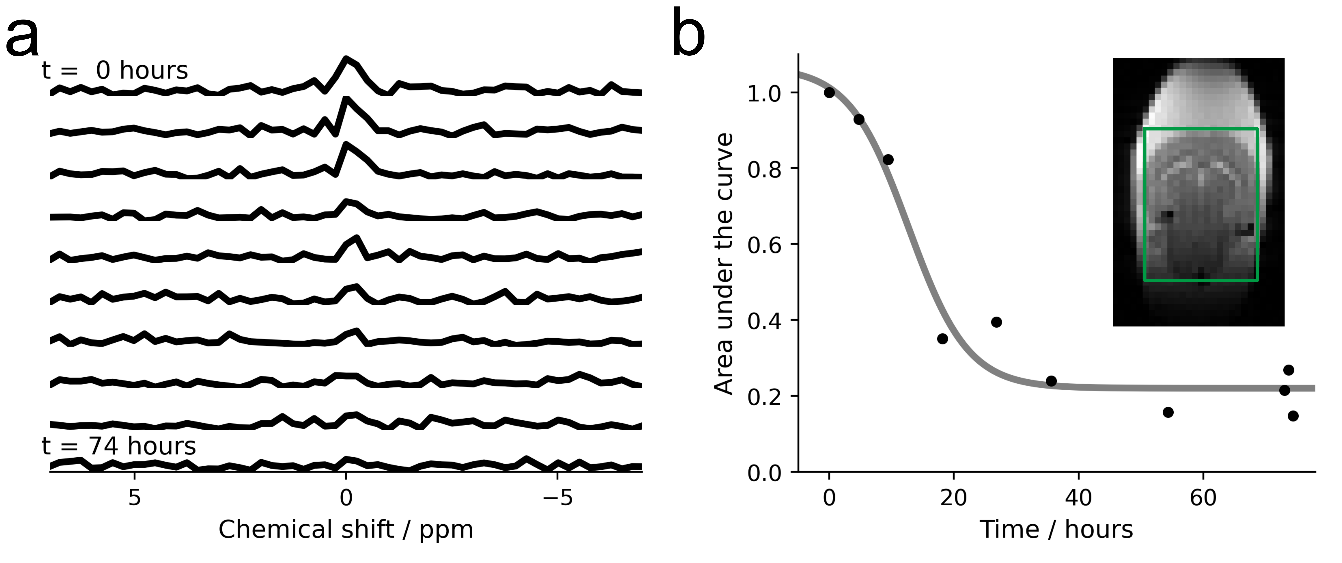


## Fitting parameters weekly ^7^Li spectra:

**Table S1:** **Fitting parameters of the weekly *in vivo* ^7^Li spectra**. The fitting parameters of the ^7^Li spectra of animal 1 and 2 (group 2) are given for each week of treatment. Due to technical problems, no measurements were possible in week 2 of treatment.

| **Week** | **Animal 1** | | | **Animal 2** | | |
| --- | --- | --- | --- | --- | --- | --- |
|  | AUC | Chemical shift / ppm | FWHM / ppm | AUC | Chemical shift / ppm | FWHM / ppm |
| 1 | 4.61±0.13 | 0.13±0.005 | 0.47±0.02 | 3.22±0.11 | 0.15±0.005 | 0.34±0.03 |
| 2 | **-** | **-** | **-** | **-** | **-** | **-** |
| 3 | 4.45±0.13 | 0.08±0.007 | 0.49±0.02 | 3.85±0.14 | 0.09±0.009 | 0.53±0.03 |
| 4 | 4.26±0.13 | 0.11±0.006 | 0.50±0.02 | 2.96±0.11 | 0.13±0.007 | 0.46±0.03 |
| 5 | 4.34±0.12 | 0.07±0.009 | 0.47±0.03 | 2.85±0.11 | 0.14±0.006 | 0.40±0.03 |

AUC = area under the curve, FWHM = full-width at half-maximum of the fitted Lorentzian curve

## Measuring T1 *in vivo*:

**Fig. S2:** Lorentzian fits of the mean ^7^Li spectra at each TR with the y-axis scaled individually. The fitting parameters are given in table S2. The ^7^Li spectra of the T1 measurement are plotted using the same y-axis across all repetition times, B. The repetition time increases down the figure.


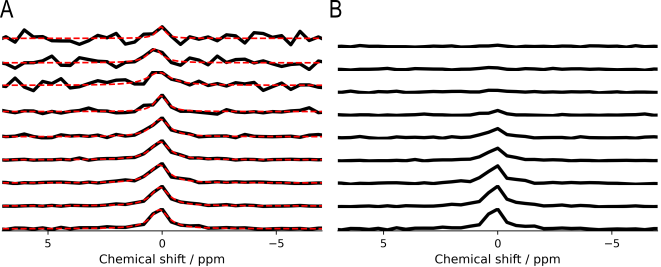


**Table S2**: *In vivo* T1 measurement of lithium-7. The fitting parameters of the normalized Lorentzian curves. The area under the curve has been normalized such that the area at TR = 40000 ms is 1. The full-width half-maximum was, as expected, larger in the non-localized spectra than in the localized spectra.

| Repetition time / ms | Normalized area  under the curve | Chemical shift / ppm | Full-width  half-maximum / ppm |
| --- | --- | --- | --- |
| 250 | 0.04±0.02 | 0.08±0.15 | 0.37±0.61 |
| 500 | 0.09±0.02 | -0.10±0.10 | 0.82±0.29 |
| 750 | 0.16±0.03 | 0.06±0.14 | 1.50±0.41 |
| 1000 | 0.16±0.02 | 0.13±0.05 | 0.60±0.10 |
| 2500 | 0.44±0.02 | 0.12±0.02 | 0.84±0.06 |
| 5000 | 0.66±0.03 | 0.11±0.02 | 0.93±0.05 |
| 10000 | 0.89±0.03 | 0.08±0.01 | 0.87±0.04 |
| 20000 | 0.99±0.03 | 0.09±0.01 | 0.84±0.03 |
| 40000 | 1.00±0.03 | 0.07±0.01 | 0.82±0.04 |
